# Intestinal Permeability in Patients Early after Kidney Transplantation Treated with Two Different Formulations of Once-Daily Tacrolimus

**DOI:** 10.3390/ijms24098344

**Published:** 2023-05-06

**Authors:** Aureliusz Kolonko, Natalia Słabiak-Błaż, Patrycja Pokora, Grzegorz Piecha, Andrzej Więcek

**Affiliations:** Department of Nephrology, Transplantation and Internal Medicine, Medical University of Silesia, 40-027 Katowice, Poland

**Keywords:** biomarkers, tacrolimus absorption, extended-release tacrolimus formulation, prolonged-release tacrolimus

## Abstract

Adequate tacrolimus blood exposure is crucial in the early post-renal transplant period and a gut epithelial barrier integrity may play a role. We prospectively investigated several markers of intestinal permeability in recent kidney transplant recipients (KTRs) treated with different tacrolimus extended-release formulations. Within each of the 49 KTR pairs that received grafts from the same donor, an early randomized conversion was performed from twice-daily (Prograf) to once-daily tacrolimus formulation: Advagraf or Envarsus. Plasma zonulin, calprotectin, circulating lipopolysaccharide (LPS), LPS-binding protein (LBP), intestinal fatty acid binding protein (FABP-2), and CD-14 levels were measured. There was no difference in the recipient age, dialysis vintage, BMI, and residual diuresis between Advagraf and Envarsus groups. FABP-2 and LPS levels were significantly associated with tacrolimus trough level, 3-h level, and area under the curve (AUC) in the Envarsus but not in the Advagraf group. AUC was independently increased by LPS and decreased by age, FABP-2 concentration, and the use of Envarsus formulation as compared with Advagraf. Functional changes of gastrointestinal tract in patients treated with Envarsus may influence intestinal tacrolimus absorption to a greater extent than in Advagraf-treated KTRs and may lead to inadequate variability of tacrolimus exposure early after kidney transplantation.

## 1. Introduction

Tacrolimus is the most common calcineurin inhibitor used in immunosuppressive regimens after kidney transplantation. Because of its high intra-patient variability and narrow therapeutic index, accurate dosing early after transplantation is still a great challenge. To date, several factors were identified which influence the individual tacrolimus metabolic rate and, subsequently, the blood trough concentration to the daily dose (C/D) ratio; they include age, body mass index (BMI), the presence of anti-HCV antibodies, and blood hemoglobin concentration [1,2]. Moreover, tacrolimus level may also be modified by interactions with other simultaneously given drugs [2,3] or disturbances of the gastrointestinal tract function, i.e., diarrhea [4]. This latter phenomenon suggests an important role for gut epithelial barrier integrity in tacrolimus absorption.

After many years of the successful clinical use of the original twice-daily tacrolimus formulation (Prograf^®^; Astellas Pharma Inc., Tokyo, Japan), two other pharmaceutic forms of tacrolimus for once-daily use were invented to increase the patient compliance and to improve the long-term transplant outcomes. There are two more-often used prolonged-release tacrolimus formulations: Advagraf^®^, manufactured by Astellas and recently developed extended-release LCPT; and Envarsus^®^, produced by Chiesi Farmaceutici S.p.A., Parma, Italy. In contrast to the twice-daily drug formulation, a substantial fraction of both these formulations is absorbed along the distal part of the gastrointestinal tract [5]. Notably, the LCPT tacrolimus has been based on the MeltDose^TM^ drug delivery technology, which was designed to enhance oral bioavailability and allow the absorption of the drug in more distal parts of the intestine [6]. In consequence, the LCPT formulation was shown to have favorable dose requirements and therapy costs in comparison to other tacrolimus formulations [7]. On the other hand, one can suspect that the absorption of both prolonged-released tacrolimus formulations could be markedly influenced by intestinal permeability. In fact, gut microbiota composition has been shown to correlate with tacrolimus dosing in kidney transplant recipients (KTRs) [8].

Until now, we have not been aware of any literature evidence concerning the potential association between intestinal permeability and tacrolimus exposure in KTRs. Hence, we performed the prospective study to analyze and compare the relationship between several markers of intestinal permeability with tacrolimus dosing and exposure in recent KTRs treated with two different formulations of prolonged-release tacrolimus.

## 2. Results

Ninety-eight KTRs (mean age, 49 ± 13 years) were recruited into this study and two parallel groups were formed after the conversion to once-daily tacrolimus formulations: patients treated with Advagraf (N = 49) or Envarsus (N = 49). The conversion was performed at median day 8 (IQR: 7–9) post-transplant. The baseline characteristics of the study groups are given in Table 1. Patients in both groups did not differ in regard to age, BMI, dialysis vintage, residual diuresis, HLA mismatch, cold ischemia time, type of induction therapy, or the occurrence of delayed graft function after transplantation. The mean age of donors was 47 ± 13 years.

As expected, the tacrolimus dose was significantly lower, whereas the tacrolimus C/D ratio was higher in the Envarsus group (Table 1) as compared with the Advagraf group. In a whole study group, the recipient’s age was negatively associated with tacrolimus daily dose (R = −0.429; *p* < 0.001) and dose per kg of body weight (R = −0.464; *p* < 0.001). There was no association between age and tacrolimus trough concentration, whereas the negative correlations were noted between age and tacrolimus 3-h level (R = −0.298; *p* < 0.01) as well as AUC (R = −0.257; *p* = 0.01). The tacrolimus C/D ratio positively correlated with recipient’s age (R = 0.344; *p* < 0.001). Importantly, tacrolimus AUC was significantly greater in the Advagraf group despite its lower trough concentration, as the tacrolimus 3h concentration was markedly higher in this group (Table 1).

**Figure 1:**
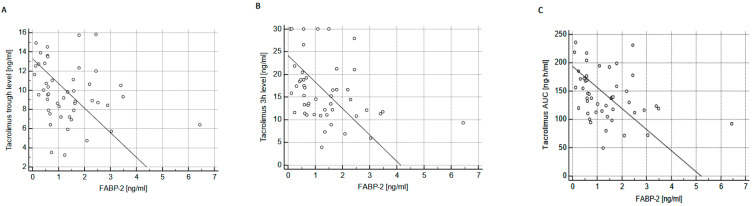
The association between FABP-2 plasma concentration and tacrolimus trough level (**A**); 3-h post-dose level (**B**); and tacrolimus area under the curve (AUC) (**C**) in patients treated with Envarsus.

In the Envarsus group, FABP-2 was inversely associated with tacrolimus trough level (R = −0.347; *p* < 0.05), 3-h level (R = −0.442; *p* < 0.01) and, consequently, with tacrolimus AUC (R = −0.419; *p* < 0.01) (Figure 1).

**Figure 2:**
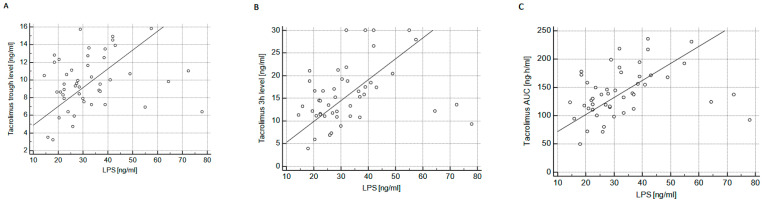
The association between LPS plasma concentration and tacrolimus trough level (**A**); 3-h post-dose level (**B**); and tacrolimus area under the curve (AUC) (**C**) in patients treated with Envarsus.

There were no similar associations in the Advagraf group (*p* = 0.46, 0.68 and 0.76, respectively). Moreover, there were positive correlations between LPS and tacrolimus trough level (R = 0.298; *p* < 0.05), 3-h level (R = 0.386; *p* < 0.01), and AUC (R = 0.410; *p* < 0.01) in the Envarsus group (Figure 2), but not Advagraf group (*p* = 0.75, 0.12 and 0.10, respectively).

Notably, there were no correlations of FABP-2 and LPS levels with tacrolimus dosing. The rest of analyzed markers of intestinal permeability showed no significant associations with tacrolimus exposure measures (Table 2).

In the whole study group, the frequency of diarrhea was low and equal in both groups (8.7%). There was no difference in maximum and discharge ALT and GGT activity between analyzed groups. Moreover, blood levels of mycophenolate mofetil were comparable [1.65 (1.4–3.1) in the Advagraf group versus 1.8 (1.0–2.7) ng/mL in the Envarsus group; *p* = 0.61)] (Table 2).

When analyzing the association between the study biomarkers panel and eGFR at the discharge, calprotectin level negatively (R = −0.242; *p* < 0.05)—whereas LBP positively (R = 0.356; *p* < 0.001)—correlated with eGFR. For zonulin level, this association was of borderline significance (R = 0.193; *p* = 0.06). BMI at the day of discharge from the hospital was associated only with LPS concentration (R = −0.248; *p* < 0.05). Of note, only calprotectin (R = 0.282; *p* < 0.01) and CD-14 (R = 0.308; *p* < 0.01) levels positively correlated with CRP, whereas we did not find any correlations of intestinal permeability markers with IL-6.

In a whole study group, the stepwise multiple regression analysis revealed that AUC was independently increased by LPS concentration (r_partial_ = 0.210; *p* < 0.05) and decreased by age (r_partial_ = −0.300; *p* < 0.01), FABP-2 concentration (r_partial_ = −0.279; *p* < 0.01), and the use of Envarsus formulation as compared with the Advagraf (r_partial_ = −0.242; *p* < 0.05). In the analogic regression analysis performed in the Advagraf group, the only independent variable influencing the tacrolimus AUC was the tacrolimus dose per kg of body weight. Of importance, this variable was deleted from the model in the Envarsus group, whereas FABP-2 level remained the only independent parameter which influenced the tacrolimus AUC (r_partial_ = −0.421; *p* < 0.01), with age (r_partial_ = −0.282; *p* = 0.06) and LPS level (r_partial_ = 0.274; *p* = 0.07) left in the statistical model with borderline significance.

## 3. Discussion

In the present prospective study, we investigated the association between intestinal permeability and tacrolimus exposure in the early post-transplant period. The paired kidney analysis was chosen to eliminate potential bias connected to donor factors. In our cohort, a multivariate regression analysis confirmed that tacrolimus AUC was independently influenced by both LPS and intestinal FABP plasma levels, as well as by the type of prolonged-release tacrolimus formulation. As the markers of intestinal permeability, i.e., LPS and FABP-2, were found among explanatory variables only in patients treated with Envarsus, but not in the Advagraf group, this could partly explain the observed differences in tacrolimus exposure between those two study groups. Thus, patients receiving Envarsus could be more prone to inadequate variability of tacrolimus exposure early after kidney transplantation. In fact, significantly lower tacrolimus AUC was found in patients treated with Envarsus as compared with those treated with Advagraf, despite higher tacrolimus trough concentrations in the former group.

Intestinal absorption is a crucial factor regulating the overall tacrolimus exposure in KTRs, especially in patients treated with the modified-release drug formulations [9]. On the other hand, increased intestinal permeability and higher endotoxin levels were found in tacrolimus-treated liver transplant patients as compared with healthy volunteers [10]; however, these effects were not confirmed by others [11]. Interestingly, distinct microbiota configurations were observed in KTRs with post-transplant diarrhea, acute rejection, and Enterococcus urinary tract infection [12]. Several biomarkers have been proposed to assess intestinal permeability in different animal models and patient populations, including zonulin [13,14,15], LPS [16,17], LBP [15,18], and FABP-2 (intestinal FABP, aka I-FABP) [19,20,21] plasma levels. Additionally, besides all above markers, we also assessed the plasma levels of the calprotectin–a marker of intestinal inflammation [22]. Fecal calprotectin concentration has also been shown to correlate with intestinal permeability [13]. Lastly, the plasma CD-14 was determined, as it was found as a specific receptor for LPS, participating in the colon permeation mechanism in the animal model [17].

Among all analyzed markers of intestinal permeability and function, we documented significant associations of FABP-2 and LPS plasma concentrations with tacrolimus AUC only in patients treated with Envarsus, but not with Advagraf. Previously, FABP-2 was identified as an early marker for diagnosing necrotizing enterocolitis in pre-term infants [20], being highly specific for mucosal damage in the small intestine [19]. Recently, increased levels of intestinal FABP were also observed in patients with type 1 and type 2 diabetes mellitus, corresponding with diabetic gut barrier dysfunction [23]. Moreover, FABP-2 was used for the calculation of a permeability risk score, together with LPS and LBP levels [21]. On the other hand, damage to the integrity of the gut epithelial barrier allows bacteria and their products, especially LPS, to translocate from the intestinal lumen into the peripheral blood [24]. LPS plays an essential role in inducing both intestinal tissue and systemic inflammatory responses [25]. LBP contributes to the transfer of LPS on innate immune cells expressing CD-14 and toll-like receptor-4, which stimulates the release of pro-inflammatory cytokines [24]. Both LPS and LBP levels were confirmed to be higher in patients with increased intestinal permeability [15,18,21]. The last analyzed marker, zonulin, was described as a physiologic modulator of intercellular tight junction function [26]. However, in the present study, zonulin level, unexpectedly, was not associated with tacrolimus exposure. In fact, recent findings have doubted its specificity as an intestinal permeability marker [27,28]. Moreover, as the tacrolimus administration was shown to induce only a selective dysfunction in transcellular monosaccharide absorption, but not paracellular permeability pathway [11], it may partly explain our results.

To date, only a few clinical studies were performed to directly compare both once-daily tacrolimus formulations in de novo KTRs, with the analysis of bioavailability and safety as the main study outcomes. As expected, LCPT has greater bioavailability [6,7,29] and lower therapy cost than Prograf and Advagraf [7]. In the present study, we found that markers of intestinal permeability were significantly associated with tacrolimus exposure measures only in patients treated with Envarsus. Consequently, those patients could be more prone to tacrolimus absorption disturbances caused by the intestinal disorders in the early post-transplant period. Importantly, the variability of trough levels and C/D ratios was previously shown to be the highest in patients treated with LCPT formulation [7]. This is in line with our present findings and may result from the greater influence of intestinal disturbances in the early post-transplant period on the overall tacrolimus absorption and exposure in LCPT-treated KTRs. It remains to be clarified if the use of novel drug release technology (MeltDose) may play a role in such interference in patients treated with Envarsus. However, except for the single report concerning the association between diarrhea and elevated tacrolimus levels, we did not find other original publications covering this topic. Importantly, the frequency of diarrhea, the results of liver function tests, and mycophenolate mofetil blood levels were comparable in both study groups. Thus, our present investigation may help to elucidate the reciprocal relationship between intestinal permeability and tacrolimus exposure in patients in the early post-kidney transplantation period.

Because of technical constraints, we did not collect the fecal specimens of the study patients, which is a limitation of this study, as the potential markers of intestinal permeability, including zonulin or calprotectin, were not determined in the stool. Another limitation is the lack of CYP3A5 polymorphism analysis in study patients. Nevertheless, to our knowledge, this is the first clinical prospective study to analyze the potential influence of intestinal permeability on the tacrolimus exposure in recent KTRs. Taking into account the essential role of adequate immunosuppression doses titration in the early post-transplant period, we might expect that the results of the present investigation would be helpful in the treatment strategy tailoring in this population.

## 4. Materials and Methods

### 4.1. Study Group

During the period between September 2019 and April 2021, we prospectively analyzed 49 consecutive pairs of KTRs who received their graft from the same donor. Within each pair of recipients, an early conversion was performed from the initial twice-daily (Prograf) to once-daily tacrolimus formulation: either Advagraf or Envarsus. At the day of discharge from the hospital, plasma concentrations of zonulin, calprotectin, circulating lipopolysaccharide (LPS), LPS-binding protein (LBP), and intestinal fatty acid binding protein (FABP-2) were measured. Tacrolimus exposure was assessed using area under the curve (AUC) based on blood trough and 3-h post-dose levels [30]. Tacrolimus C/D ratio was calculated based on the last tacrolimus blood trough level before the discharge. The study was conducted in concordance with the protocol of Helsinki. The Bioethical Committee of the Medical University of Silesia accepted the study protocol (No. KNW/0022/KB1/81/18), and all participants gave their written informed consent.

### 4.2. Immunosuppression Protocol

Initial immunosuppressive protocol consisted of twice-daily tacrolimus (Prograf), mycophenolate mofetil (750 mg BID) and steroids. In all patients, induction therapy using basiliximab or rabbit antithymocyte globulin (rATG) was administered. First doses of tacrolimus and mycophenolate were given pre-operatively. Steroids were given intravenously during operation (500 mg), than 125 mg i.v. the next day, and subsequently 20 mg of oral prednisolone daily, with further dose reduction. Patients receiving rATG (up to 7 days post-transplantation) were premedicated before each dose with 125 mg of methylprednisolone instead of prednisolone. After the second tacrolimus measurement and dose adjustment, patients were converted to Advagraf or Envarsus in the randomized single-blinded manner within each pair of subjects, who each received their kidney grafts from the same donor.

### 4.3. Laboratory and Hormonal Parameters

Biochemical parameters (serum creatinine and C-reactive protein (CRP) levels, alanine aminotransferase (ALT), and gammaglutamyltranspeptidase (GGT) activity) were measured using standard laboratory methods. Kidney graft function was assessed using an estimated glomerular filtration rate (eGFR) calculated according to the Modification of Diet in Renal Disease formula.

Plasma concentrations of zonulin and calprotectin (Immundiagnostik AG, Bensheim, Germany) were assessed using commercially available ELISA kits, with the intra-assay and inter-assay coefficients of variability being <5 and <8.5% and <3.3 and <9.0%, respectively. Plasma levels of IL-6, CD-14 (R&D Systems, Minnesota, MN, USA), and bacterial lipopolysaccharides (Uscn Life Sciences Inc., Wuhan, China) were assessed by ELISA, with the intra-assay and inter-assay coefficients of variability being <7.2 and <7.8%, <6.4 and <7.4%, and <10 and <12%, respectively. Plasma FABP-2 (R&D systems, Inc., Minneapolis, MN, USA) and LBP levels (CloudClone Corp, Katy, TX, USA) were measured by ELISA kit with the intra-assay and inter-assay coefficients of variability being <4.1 and 11.1% and <10 and <12%, respectively.

### 4.4. Data and Statistical Analysis

Delayed graft function was defined as a need for dialysis therapy in the first post-transplant week.

Statistical analyses were performed using Statistica 13.3 PL for Windows (Tibco Inc., Palo Alto, CA, USA) and MedCalc v19.2.1 (MedCalc Software, Mariakerke, Belgium). Values are presented as means with 95% confidence interval, medians with Q1–Q3 values, or frequencies. The main study comparison was performed between groups of patients treated with Advagraf or Envarsus prior to discharge, using the Student *t* test (for quantitative variables) or the χ2 test (for qualitative variables). Variables with non-normal distribution were compared using the Mann–Whitney U test. Correlation analyses were performed using the Spearman rank test. In a whole study group, stepwise multiple regression analysis was performed for the tacrolimus AUC as a dependent variable and age, BMI, tacrolimus dose per kg of body weight, FABP-2 and LPS concentrations, and the use of Envarsus versus Advagraf formulation as potential independent variables. We also performed two analogic analyses in separate groups of patients treated with Advagraf or Envarsus. For all analyses, a *p* value below 0.05 was considered statistically significant.

## 5. Conclusions

In summary, we found that tacrolimus exposure in recent KTRs is independently correlated with both LPS and intestinal FABP plasma concentrations. The risk factors for tacrolimus underexposure were identified separately for patients treated with both formulations of once-daily tacrolimus. Notably, a significant relationship between measures of intestinal permeability and tacrolimus exposure was noted only in the Envarsus group. Our findings suggest that functional changes in the gastrointestinal tract in patients treated with Envarsus may influence intestinal tacrolimus absorption to a greater extent than in Advagraf-treated KTRs and may lead to inadequate variability of tacrolimus exposure early after kidney transplantation.

## Figures and Tables

**Table 1:** Baseline characteristics of patients treated with Advagraf or Envarsus tacrolimus formulation. * medians with Q1–Q3 values.

	Advagraf N = 49	Envarsus N = 49	*p*
Patient			
Age [years]	52 (39–59)	50 (42–58)	0.97
Sex [M/F]	28/21	28/81	1.00
BMI [kg/m^2^]	25.2 (22.9–28.9)	25.9 (23.3–29.0)	0.51
Dialysis vintage [months] *	31 (20–44)	31 (24–50)	0.58
Residual diuresis [mL] *	500 (100–2000)	500 (150–1000)	0.96
Pre-transplant diabetes [n (%)]	6 (12.2)	6 (12.2)	1.0
Early post-transplant diabetes [n (%)]	2 (4.1)	2 (4.1)	1.0
Transplant procedure			
Retransplant [n, %]	6/43	3/46	0.30
HLA class I mismatch *	2.49 (2.25–2.73)	2.31 (2.04–2.57)	0.31
HLA class II mismatch *	0.65 (0.50–0.80)	0.61 (0.46–0.77)	0.70
Cold ischemia time [h]	17.2 (15.5–18.9)	18.9 (17.2–20.7)	0.14
Induction therapy			
Basiliximab [n, %]	35 (71.4)	33 (67.3)	0.66
Antithymocyte globulin [n, %]	14 (28.6)	16 (32.7)	
Delayed graft function [n, %]	10 (20.4)	9 (18.4)	0.80
Tacrolimus dosing and exposure prior to discharge			
Tacrolimus dose [mg/d] *	7.5 (6.0–11.0)	4.75 (3.25–7.0)	<0.001
Tacrolimus dose per kg [mg/kg] *	0.12 (0.08–0.16)	0.07 (0.05–0.10)	<0.001
Tacrolimus trough level [ng/mL] *	8.4 (7.5–9.6)	9.5 (7.9–11.6)	<0.05
Tacrolimus 3h after dose [ng/mL] *	20.8 (16.9–23.7)	14.5 (11.4–18.8)	<0.001
Tacrolimus AUC [ng·h/mL] *	157.6 (134.3–171.6)	137.1 (114.6–170.8)	<0.05
Tacrolimus C/D ratio *	1.17 (0.73–1.60)	1.98 (1.29–3.02)	<0.001

**Table 2:** Kidney graft function, inflammatory markers, liver function tests, and measures of intestinal permeability in patients treated with Advagraf or Envarsus tacrolimus formulation.

	Advagraf N = 49	Envarsus N = 49	*p*
eGFR [mL/min/1.73 m^2^]	50.4 (33.6–70.3)	53.5 (39.4–70.0)	0.88
C-reactive protein [mg/L]	3.1 (1.0–5.0)	3.3 (1.3–7.0)	0.31
Interleukin-6 [pg/mL]	3.2 (2.1–5.1)	4.2 (2.5–7.8)	0.13
ALT max [IU/L]	34 (24–54)	35 (19–55)	0.48
ALT at discharge [IU/L]	28 (17–40)	25 (17–36)	0.71
GGT max [IU/L]	54 (26–94)	48 (29–103)	0.91
GGT at discharge [IU/L]	42 (23–85)	37 (29–56)	0.91
Zonulin [ng/mL]	6.2 (5.4–7.1)	6.6 (5.2–7.6)	0.22
Calprotectin [μg/mL]	1.73 (1.37–2.61)	1.91 (1.62–3.06)	0.16
LPS [ng/mL]	29.0 (22.5–36.0)	29.0 (22.5–39.0)	0.87
LBP [μg/mL]	5.07 (3.00–7.03)	4.50 (3.62–6.55)	0.76
FABP-2 [ng/mL]	1.42 (0.83–2.05)	1.15 (0.59–1.79)	0.15
CD-14 [pg/mL]	1.68 (1.47–1.83) × 10^6^	1.51 (1.36–1.8) × 10^6^	0.24

## Data Availability

The data presented in this study are available on reasonable request from the corresponding author.
